# Rare magnetic resonance imaging findings of intracranial solitary fibrous tumor: A case report

**DOI:** 10.1097/MD.0000000000031355

**Published:** 2022-10-21

**Authors:** Zhicheng Huang, Dongqin Dai, Guangcai Tang

**Affiliations:** a Department of Radiology, Affiliated Hospital of Southwest Medical University, Luzhou, Sichuan, China.

**Keywords:** case report, hemangiopericytoma, magnetic resonance imaging, solitary fibrous tumor

## Abstract

Solitary fibrous tumor (SFT) is a rare mesenchymal spindle cell tumor. SFT in the central nervous system (CNS) is rare. This case reports rare magnetic resonance imaging (MRI) findings of intracranial SFT (ISFT).

**Patient concerns:**

We report a 47-year-old female patient who was found to have weakness in her right limb with walking instability 2 months before the visit.

**Diagnoses:**

Based on imaging, the provisional diagnosis was meningioma. Postsurgical histopathological diagnosis confirmed World Health Organization (WHO) grade I SFT/hemangiopericytoma (HPC).

**Interventions:**

The lesion was totally excised. The patient improved remarkably after the operation, without any signs of associated limb movement disorder.

**Outcomes:**

No local recurrence or metastases were observed in the follow-up 3 months after the surgery.

**Lessons:**

Intracranial SFT is a rare mesenchymal tumor. Due to different tumor components, imaging manifestations are diverse and lack of characteristics, so preoperative diagnosis is challenging. Our case provides a rare ISFT with significantly decreased signal intensity in T2 weighted images (T2WI), which is an important supplement to the rare imaging findings of intracranial SFT.

## 1. Introduction

Solitary fibrous tumor (SFT) is a rare spindle cell tumor of mesenchymal origin. It occurs most frequently in the pleura and peritoneum, but it can also occur in other organs and tissues outside the pleura.^[1]^ Since 1996, SFT of the central nervous system (CNS) was first reported by Carneiro et al, although more and more intracranial SFT (ISFT) have been reported so far, its imaging manifestations often lack specificity.^[2]^ Reports of ISFT with significantly decreased signal intensity in T2 weighted images (T2WI) sequence of magnetic resonance imaging (MRI) scan are very rare. The 2007 edition of the Central Nervous System Tumor Classification considers that SFT and hemangiopericytoma (HPC) are 2 different types of tumors. Due to the development of gene detection and immunohistochemistry in recent years, it has been found that SFTs and HPCs have similar morphological characteristics, and the 12q13 and NAB2-signal transducer and activator of transcription 6 (STAT6) fusion genes have been reversed pathologically and immunohistochemically, resulting in nuclear STAT6 expression. The diagnosis of the 2 often overlaps.^[3]^ Therefore, in 2016, the World Health Organization (WHO) combined SFTs and HPCs of the CNS into SFTs/HPCs, and divided them into 3 categories according to the cell density in the tumor, collagen content, “antler” blood vessel and cell mitotic rate, etc. Therefore, HPC is rarely used alone nowadays. The term “HPC phenotype” is now regarded as a more malignant phenotype of SFT, often representing grades II and III of SFTs/HPCs, while grade I is more inclined to the term “SFT phenotype,” and clinical behavior is usually benign.^[4]^ This combined diagnosis greatly reduces the misdiagnosis rate of SFTs and HPCs.^[3]^

## 2. Case presentation

**Figure 1.**
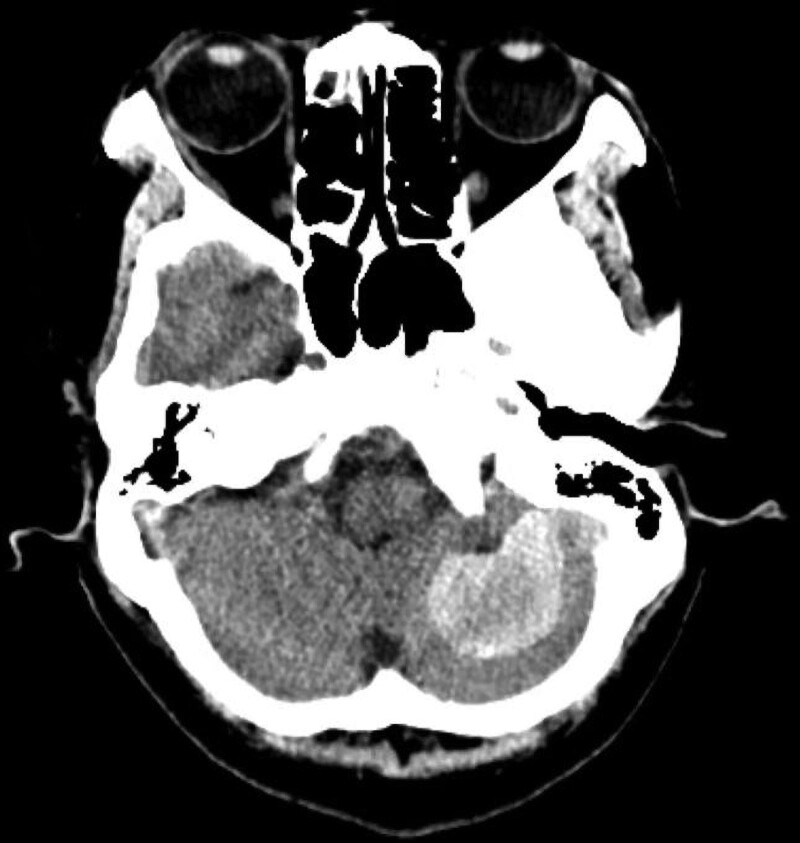
Axial head CT revealed a 3.4 cm × 2.5 cm well-defined slightly hyperdense mass without peritumoral edema in the left cerebellar hemisphere. CT = computed tomography.

**Figure 2.**
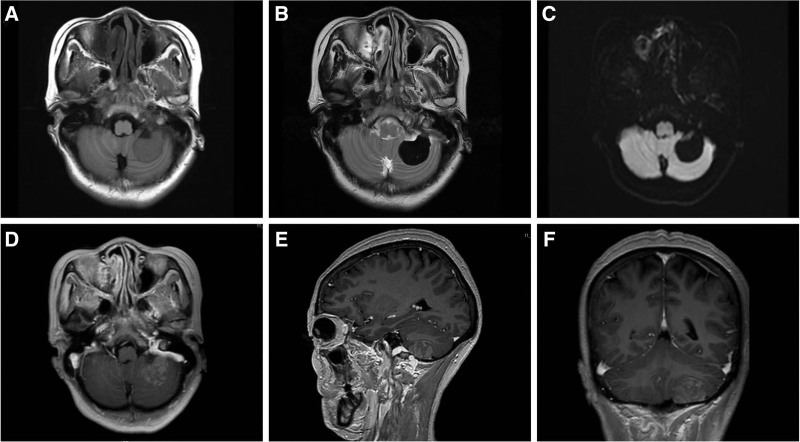
The brain MRI demonstrated a 3.0 cm × 2.1 cm × 2.1 cm ovoid mass in the left occipital. The tumor showed intermediate-slightly decreased signal intensity in T1WI (A) and significantly decreased signal intensity in T2WI (B). Axial (C) DWI did not show diffusion restricted areas. Axial (D), sagittal (E), and coronal (F) enhanced T1WI showed heterogeneous and strong enhancing tumor, connecting with the adjacent meninges by a wide base and compressing the left cerebellar hemisphere. DWI = diffusion weighted imaging, MRI = magnetic resonance imaging, T1WI = T1 weighted images, T2WI = T2 weighted images.

A 47-year-old female patient found that her right limb was weak and her walking was unstable 2 months before the visit. The patient has a history of hypertension and no history of trauma, surgery or other diseases. Computed tomography of the head showed 3.4 cm × 2.5 cm well-defined slightly hyperdense mass without peritumoral edema in the left cerebellar hemisphere (Fig. 1). The brain MRI demonstrated a 3.0 cm × 2.1 cm × 2.1 cm ovoid mass in the left occipital. The tumor showed intermediate-slightly decreased signal intensity in T1 weighted images (T1WI) (Fig. 2A) and significantly decreased signal intensity in T2WI (Fig. 2B). Diffusion-weighted imaging did not reveal areas with diffusion restriction (Fig. 2C). On enhanced T1WI, the tumor showed uneven enhancement (Fig. 2D–F), and the tumor was connected with the adjacent meninges by a wide base, compressing the left cerebellar hemisphere. According to the clinical and imaging findings, it was initially diagnosed as meningioma.

**Figure 3.**
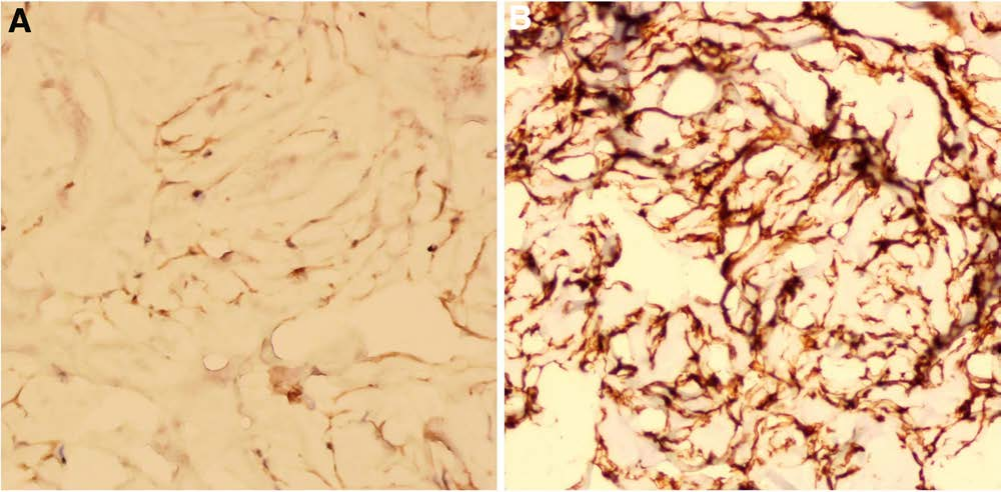
The cells were strongly positive for STAT6 (A) and Vim (B), negative for EMA, S-100, GFAP and PR, Ki-67 (<1%). EMA = epithelial membrane antigen, GFAP = glial fibrillary acidic protein, PR = progesterone receptor, STAT6 = signal transducer and activator of transcription 6, Vim = vimentin.

The tumor was surgically removed. The surgical results showed that the mass adhered closely to the dura mater at the left petrosal bone and had a clear border with the left cerebellar hemisphere. In addition, biopsy was performed after tumor resection. The cells were strongly positive for STAT6 (Fig. 3A) and vimentin (Fig. 3B), negative for epithelial membrane antigen (EMA), S100, glial fibrillary acidic protein and progesterone receptor, Ki-67 (<1%). The immunohistochemical characteristics of our case strongly suggest SFT, and according to the latest WHO classification, it is diagnosed as Grade I. Patient recovered and discharged. There was no evidence of local recurrence or metastasis in the follow-up 3 months after operation. However, due to the short follow-up time and uncertain prognosis, it is recommended that the patient return every 3 months for further follow-up.

## 3. Discussion

ISFT is a rare mesenchymal tumor, which originates from CD34 positive dendritic mesenchymal cells and is named for its differentiation into fibroblasts and myofibroblasts. ISFT is a borderline tumor, accounting for <1% of all primary CNS tumors, most of which are benign, and 10% to 20% are malignant or potentially malignant.^[5]^ SFTs most often occur in the pleura and peritoneum, but also in other tissues and organs outside the pleura. ISFT is rare, accounting for about 11% of systemic SFT, and is common in adults aged 40 to 60 years.^[6]^ The symptoms and signs of ISFT are mainly related to the location, size, benign and malignant of tumors.^[7]^ ISFT usually occurs at the skull base, near the cerebral falx, sagittal sinus and tentorium cerebelli, and it has also been reported that ISFT occurs in the ventricles of the brain.^[8]^ When the tumor becomes large enough or invades important functional areas, patients will have clinical symptoms, including paroxysmal headache, dizziness, gait imbalance, sensory disturbance, hemiplegia and seizures.^[9,10]^ WHO Grade I of intracranial SFTs/HPCs often produces local compression symptoms through slowly growing tumors, while WHO grade II and grade III are more manifested as biological behaviors of malignant tumors, such as adjacent bone invasion and destruction, regional brain nerve function damage and metastasis, etc.

Radiological features are helpful to predict tumor pathology, such as T2WI signal intensity of tumor solid components, which is helpful to evaluate who grading of Pathology.^[11]^ The low signal intensity on T2WI may be caused by the densely arranged collagen fibers in the tumor, while the high signal intensity mostly represents the tumor rich cell area. For WHO grade I, most cases showed isointense or low signal intensity on T2WI.^[12]^ Some cases may have map like changes of alternating high and low signal areas on T2WI, which we call “Yin Yang sign.” while low signal areas on T2WI may have enhanced high signal performance on enhanced scanning, which is called “black and white inversion.” “Yin Yang sign” and “black and white inversion” can be regarded as the characteristic imaging performance of intracranial SFTs/HPCs.^[13]^ In our case, the T2WI signal intensity of the mass decreased significantly overall. We speculate that this may be because the tumor belongs to WHO grade I, the tumor body is extremely rich in collagen fibers and widely distributed, and the tumor cell rich area also has collagen fibers to varying degrees. Therefore, it is difficult to reveal the high signal in the simple tumor cell rich area on T2WI, which ultimately leads to the significantly decreased signal intensity change of the tumor on T2WI. In our case, the tumor showed uneven and obvious enhancement on T1WI enhanced scan, and the typical “black and white inversion” sign appeared. For WHO grade II and III, tumor T2WI usually shows medium and high signal intensity. In addition, empty blood vessel shadow caused by a large number of staghorn like and fissure like blood vessels can often be seen inside or on the surface of the tumor, which is crucial to distinguish WHO grade I from WHO grade II and grade III.^[11]^ In addition to the different signals on T2WI, tumor morphology, whether the tumor is associated with hemorrhage and necrosis, whether there is peritumoral edema and adjacent bone destruction are also commonly used to predict WHO grading. In our case, the tumor has clear boundary, non lobulated shape, no hemorrhage and necrosis, no edema around the tumor, no adjacent bone infiltration and destruction, and is connected to the dura mater in a wide basal manner. These imaging manifestations provide good evidence for the diagnosis of WHO grade I, although they lack absolute correspondence with pathology. In addition to WHO classification, attention should also be paid to the differential diagnosis between ISFT and other tumors. Common intracranial tumors that need to be differentiated include fibromeningioma, schwannoma, gliosarcoma, hemangioblastoma, neurofibroma and lymphoma.^[14]^ ISFT is most easily misdiagnosed as meningioma and schwannoma, so is our case. In terms of imaging, meningioma is more common than SFT/HPC in dural tail sign and calcification, and the enhancement is more uniform, and adjacent bone often has hyperplasia reaction; On the contrary, SFT/HPC has more necrosis, cystic degeneration and vascular void effect, and the adjacent bone is mostly damaged by absorption. At the same time, Kanazawa et al believed that the ADC value can also be used as a reference to distinguish between angiomatous meningioma and SFT/HPC, and the average ADC value of angiomatous meningioma is higher.^[15]^ When meningioma and characteristic changes of SFT/HPC occur at the same time, we should be alert to the possibility of tumor collision, although this is very rare.^[5]^ Since the tumor occurs in the cerebellopontine region, which is rich in nerves, schwannomas that are prone to occur in the cranial nerve pathway should also be included in the differential diagnosis. Schwannoma originates from Schwann cells of myelinated nerve fibers and grows along the nerve sheath.^[16]^ Neurilemmoma often has cystic changes and necrosis. The MRI signal is usually uneven, usually showing long T1 and long T2 signals, and there is no empty vessel shadow.^[11]^

In general, imaging manifestations and tumor pathology often lack an absolute correspondence, and even the typical imaging manifestations of a lesion are often misleading.^[1]^ Therefore, tissue biopsy and immunohistochemistry are still the gold standard for final diagnosis. Histologically, SFT phenotype mimics fibromeningioma, while HPC phenotype mimics high-grade meningioma or sarcoma.^[5]^ The clinical behavior of tumors with SFT phenotype is usually benign, while the clinical behavior of tumors with HPC phenotype is malignant, and has a high recurrence rate and metastasis rate.^[17,18]^ Although CD34 and EMA have been used to differentiate SFT from meningioma; However, CD34 and EMA lack high sensitivity and specificity. CD34 is usually positive for SFTs/HPCs, but weakly positive in 15% to 60% of meningiomas.^[19,20]^ The positive rate of EMA in meningiomas is as high as 89.7%, so EMA is widely considered as a useful marker for the diagnosis of meningiomas.^[21]^ However, it may also be positive in 0-20% SFTs/HPCs.^[20,22]^ Therefore, detection of nuclear expression of STAT6 has been highly valued at present, because compared with these markers, the sensitivity and specificity of STAT6 to SFT can reach 96% and 100% respectively.

Compared with radiotherapy and chemotherapy, surgical resection is the most effective method to treat patients with SFTs/HPCs, and the prognosis of patients directly depends on the degree of resection.^[11]^ In the CNS, the prognosis of high-grade SFT/HPC is poor, the recurrence rate exceeds 75% 10 years later, and 21.3% of them have extracranial metastasis.^[18]^ Fargen et al’s retrospective study of 189 patients with SFTs showed that the tumor recurrence rate after total resection was significantly lower than that after subtotal resection.^[3]^ Therefore, removing tumor as much as possible is the greatest desire of SFT surgery. Our patients also adopted the method of total tumor resection. Radiotherapy after surgical resection of SFTs//HPCs is still controversial. Most neurosurgeons and neurooncologists advocate adjuvant radiotherapy, especially when the tumor is not completely removed.^[23]^ However, some scholars believe that adjuvant radiotherapy cannot improve the prognosis of patients.^[3]^ At present, no conclusions can be drawn on the effectiveness of these measures. Even if there is no research showing the effectiveness of these measures, most clinicians advocate that it is beneficial to perform customized maximum tumor resection at the time of initial surgery.^[11]^

To sum up, intracranial SFT/HPC is a rare mesenchymal tumor, lacking typical imaging features, and its diagnosis and differential diagnosis are difficult. Our case is an important supplement to the rare MRI findings of low-grade ISFT.

## Author contributions

**Conceptualization:** Dongqin Dai.

**Formal analysis:** Zhicheng Huang.

**Investigation:** Zhicheng Huang, Dongqin Dai.

**Methodology:** Zhicheng Huang.

**Supervision:** Guangcai Tang.

**Writing – original draft:** Zhicheng Huang.

**Writing – review & editing:** Guangcai Tang.
